# Downregulation of serum exosomal miR‐320d predicts poor prognosis in hepatocellular carcinoma

**DOI:** 10.1002/jcla.23239

**Published:** 2020-03-03

**Authors:** Wenxiao Li, Xuemei Ding, Shaohong Wang, Li Xu, Tao Yin, Shuangxi Han, Jianli Geng, Wenbing Sun

**Affiliations:** ^1^ Department of Hepatobiliary Surgery Beijing Chao‐yang Hospital Capital Medical University Beijing China; ^2^ Department of Hepatobiliary Surgery The Municipal Hospital of Weihai Weihai China

**Keywords:** diagnosis, hepatocellular carcinoma, miR‐320d, prognosis, serum exosome

## Abstract

**Background:**

MicroRNAs (miRNAs) is a class of functional regulator of tumorigenesis of human cancer including hepatocellular carcinoma (HCC). However, the potential clinical significance of serum exosomal miR‐320d in HCC has not been elucidated.

**Methods:**

Real‐time reverse transcription PCR was used to detect the expression pattern of serum exosomal miR‐320d in patients with HCC, and the correlation between the deregulation of serum exosomal miR‐320d and the clinical outcome of HCC was explored. The biological function of exosomal miR‐320d in HCC was also investigated.

**Results:**

Our results showed that the expression levels of exosomal miR‐320d were remarkably reduced in the serum samples of HCC patients and the culture medium of HCC cell lines compared with their respective controls. Serum exosomal miR‐320d could differentiate the HCC patients from healthy controls with high accuracy. In addition, its level was remarkably increased in the HCC patients who had received surgical treatment. Moreover, reduced serum exosomal miR‐320d was associated with advanced tumor stage, positive lymph node metastasis, and poorly differentiated tumors. HCC patients with lower serum exosomal miR‐320d had shorter overall and disease‐free survival. Low serum exosomal miR‐320d was identified to be an independent unfavorable prognostic factor for HCC. Finally, overexpression of miR‐320d inhibited the proliferation and invasion of HCC cells, and BMI1 was demonstrated to be a direct target of miR‐320d.

**Conclusion:**

Taken together, serum exosomal miR‐320d could be a potential non‐invasive biomarker for the diagnosis and prognosis of HCC.

## 1 INTRODUCTION

Hepatocellular carcinoma (HCC) is one of the most commonly occurring malignant cancers in the world, with high morbidity and mortality.^1^ Due to the lack of featured signs or symptoms in the early stages, patients with HCC are often confirmed at the late stage.^2^ Metastasis is the major reason responsible for the unfavorable clinical outcome and death of HCC.^3^ The initiation and progression of HCC involves in loss of tumor suppressor genes and activation of oncogenes. However, currently the underlying molecular mechanisms are poorly known. Therefore, exploring effective biomarkers with high specificity and sensitivity for HCC diagnosis and prognosis as well as illustrating the molecular mechanism of HCC are urgently needed.

MicroRNAs (miRNAs) are evolutionary conserved short non‐coding RNAs with 19‐25 nucleotides in length.^4^ These small molecules regulate gene expression at the post‐transcriptional level by binding to the seed sequences in the 3′‐untranslated region (3′‐UTR) of target mRNAs, leading to destabilization/degradation of mRNA and/or inhibition of translation. miRNAs have been shown to play key roles in regulating many biological processes such as proliferation, differentiation, apoptosis, migration, and invasion.^5^ Not surprisingly, aberrant miRNA expression is closely implicated in the onset and progression of various types of cancers including HCC.^6^, ^7^, ^8^ For instance, the expression level of miR‐543 was upregulated in HCC tissues. In addition, overexpression of miR‐543 promoted the proliferation, migration, and invasion of hepatocellular carcinoma cells by targeting PAQR3, suggesting miR‐203a‐3p played an oncogenic role in HCC development.^9^

Exosomes are membrane‐derived nanovesicles with 30‐100 nm in diameter.^10^ They are important for cell‐to‐cell communication and can be secreted into the biofluids such as serum, plasma, saliva, and urine.^11^ Exosomes contain various molecular constituents of their cell of origin including proteins, mRNA, non‐coding RNA, and DNA.^12^ A line of evidence has demonstrated that circulating exosomal miRNAs control the initiation, progression, and metastasis of cancers including HCC, and they show great promise as non‐invasive biomarkers for the early detection and prognosis prediction of cancer.^13^, ^14^, ^15^

Our preliminary study has profiled the expression levels of serum exosomal miR‐320 family (miR‐320a, miR‐320b, miR‐320c, miR‐320d, and miR‐320e) in HCC patients and healthy controls with a small sample size. The circulating exosomal miR‐320a and miR‐320d levels were significantly lower in HCC patients than in healthy volunteers. However, no significant difference was found for serum exosomal miR‐320b, miR‐320c, and miR‐320e levels between HCC patients and healthy controls (data not shown). Serum exosomal miR‐320d was more consistently downregulated in HCC patients than serum exosomal miR‐320a, and thus, it was selected as the candidate biomarker for further investigation. In the present study, we aimed to elucidate the diagnostic and prognostic value of serum exosomal miR‐320d in HCC. Our results showed that the expression level of circulating exosomal miR‐320d was reduced in patients with HCC. In addition, low serum exosomal miR‐320d was significantly associated with unfavorable clinicopathological parameters and worse prognosis. Mechanistically, ectopic expression of miR‐320d suppressed the proliferation and invasion of HCC cells by targeting BMI1.

## 2 MATERIALS AND METHODS

### 2.1 Patients and sample collection

The study protocol was approved by the Institutional Review Board of the Beijing Chao‐yang Hospital, and all participants provided informed consent. A total of 110 patients with newly diagnosed HCC and 40 healthy volunteers were recruited in this study. None of the patients received surgery, chemotherapy, or radiotherapy before first‐time serum sample collection. All patients with HCC were pathologically confirmed by two trained pathologists. The tumor stage was evaluated based on the American Joint Committee on Cancer staging system. The clinicopathological information such as age, gender, tumor size, tumor stage, lymph node metastasis, and distant metastasis was collected and summarized in Table 1. The healthy volunteers were randomly selected from an early HCC screening project, and they were matched to the HCC cases by age and gender. All the participants were Han Chinese.

**Table 1:** The association between serum exosomal miR‐320d and clinicopathological parameters of HCC

Parameters	N (%)	Serum exosomal miR‐320d		*P*
		Low (n = 57)	High (n = 53)	
Gender				
Male	98 (89.09)	51 (46.36)	47 (42.73)	.8938
Female	12 (10.91)	6 (5.45)	6 (5.45)	
Age				
≤60 y	47 (42.73)	26 (23.64)	21 (19.09)	.5256
>60 y	63 (57.27)	31 (28.18)	32 (29.09)	
Tumor size				
<5 cm	79(71.82)	38(34.55)	41(37.27)	.2130
≥5 cm	31 (28.18)	19 (17.27)	12(10.90)	
AFP				
<400 ng/mL	40 (36.36)	25 (22.73)	15(13.64)	.0901
≥400 ng/mL	70 (63.64)	32 (29.09)	38 (34.55)	
Differentiation				
Well/Moderate	74(67.27)	33(30.00)	41 (37.27)	.0297
Poor	36 (32.73)	24 (21.82)	12 (10.90)	
LNM				
Negative	68(61.82)	28(25.45)	40(36.36)	.0045
Positive	42 (38.18)	29 (26.36)	13 (11.82)	
Metastasis				
Negative	105 (95.45)	53 (48.18)	52 (47.27)	.1968
Positive	5 (4.54)	4 (3.64)	1 (0.9)	
TNM stage				
I‐II	64 (58.18)	26 (23.64)	38 (34.55)	.0056
III‐IV	46 (41.82)	31 (28.18)	15 (13.64)	

At least 5 mL of peripheral blood sample was collected from each participant. The serum specimens were obtained by centrifuging the blood samples at 2000 rpm for 15 minutes and 11 000 rpm for 5 minutes within 30 minutes of collection. Then, the serum samples were aliquoted and stored at −80°C until further analysis.

### 2.2 Exosome isolation and qRT‐PCR

Exosomes were isolated from serum/culture medium using the total exosome isolation reagent (Invitrogen) according to the manufacture's protocols. Total Exosome RNA and Protein Isolation Kit (Invitrogen) and miRNeasy mini kit (QIAGEN) were used to isolate RNA from exosome or cells, respectively. The cDNA was synthesized with PrimeScript^™^ RT Master Mix (Takara Biotechnology Co. Ltd), and quantitative real‐time PCR was conducted using SYBR Premix Ex Taq II (Takara Biotechnology) in CFX96TM Real‐Time PCR Detection System (Bio‐Rad Laboratories Inc). The relative expression of exosomal miRNAs or mRNAs was normalized to the internal control genes U6 snRNA or GAPDH, respectively, and calculated using the 2^−ΔΔ Ct^ method.

### 2.3 Cell culture and transfection

The HCC cell lines HepG2, SNU‐449, Hep3B, Huh‐7, SNU‐423, and SNU‐387 and normal hepatic cell line L02 were maintained in DMEM (Invitrogen) supplemented with 10% fetal bovine serum (Invitrogen), 100 U/mL penicillin, and 100 mg/mL streptomycin. The cells were cultured in a humidified incubator at 37°C with 5% CO_2_. Cells were transfected with miR‐320d mimics or scrambled miRNA controls preincubated with exosomes with Lipofectamine RNAiMAX transfection reagent (Invitrogen) according to the manufacturer's instructions.

### 2.4 CKK‐8 assay

The miR‐320d overexpression HCC cells and the control cells were plated into 96‐well plates at a density of 3000 cells per well. The effect of miR‐320d upregulation on the proliferation of HCC cells was determined at 0, 24, 48, 72, and 96 hours after incubation using the Cell Counting Kit‐8 (CCK‐8; Dojindo Laboratories). The optical density (OD) value was examined using a spectrophotometer (Bio‐Rad Laboratories Inc) at a wavelength of 450 nm.

### 2.5 Invasion assay

The cell invasion assay was performed with the Matrigel‐coated Transwell cell culture chamber (8 μm pore size; Corning Incorporated, Life Sciences). The miR‐320d overexpression HCC cells or the control cells (1 × 10^5^ cells) were seeded into the upper chamber in the pure DMEM, and the lower chamber was filled with completed culture medium. Following 24 hours of incubation, the cells remaining at the upper chamber were removed with a cotton swab. The cells at the lower surface of the chamber were fixed with 4% formaldehyde and stained with crystal violet. The number of invaded cells was counted under the microscope.

### 2.6 Western blotting

The exosomal proteins were resolved by SDS‐PAGE and transferred to PVDF membranes (Millipore). The membranes were blocked with 5% milk in TBST for 1 hour at room temperature and then incubated with primary antibodies against TSG101 and CD63 overnight at 4°C. After washing, the membrane was incubated with HRP‐conjugated secondary antibodies for 1 hour at room temperature. Proteins were visualized using Amersham ECL Prime Western Blotting Detection Reagent (Amersham Biosciences).

### 2.7 Luciferase reporter assay

The binding site was cloned into psiCHECK2 vector to form the reporter vector psiCHECK2‐BMI1 wild type. The psiCHECK2‐BMI1 was co‐transfected with miR‐320d mimics or scrambled miRNA controls into HCC cells by Lipofectamine 2000 (Invitrogen) according to the manufacturer's instruction. After 48 hours of transfection, the relative luciferase activity was evaluated using the Dual‐Glo Luciferase Assay Kit (Promega).

### 2.8 Statistically analysis

Statistical analyses were performed using the GraphPad Prism 7 (GraphPad Software, Inc) and MedCalc 14.8.1 (MedCalc Software). The difference between two groups was analyzed by two‐tailed Student’s *t* test or Mann‐Whitney *U* test. The difference among more than two groups was analyzed with one‐way ANOVA. Receiver‐operating characteristic (ROC) curves and area under the curve (AUC) were used to examine the diagnostic value of serum exosomal miR‐320d. The association between serum exosomal miR‐320d and the clinicopathological parameter was evaluated by the Chi‐square test. The Kaplan‐Meier method and log‐rank test were used to assess overall survival (OS) and disease‐free survival (DFS). Multivariate Cox regression analysis was performed to identify the independent prognostic factors for HCC *P < *.05 was considered as statistically significant.

## 3 RESULTS

### 3.1 Serum exosomal miR‐320d was significantly reduced in patients with HCC

**Figure 1:**
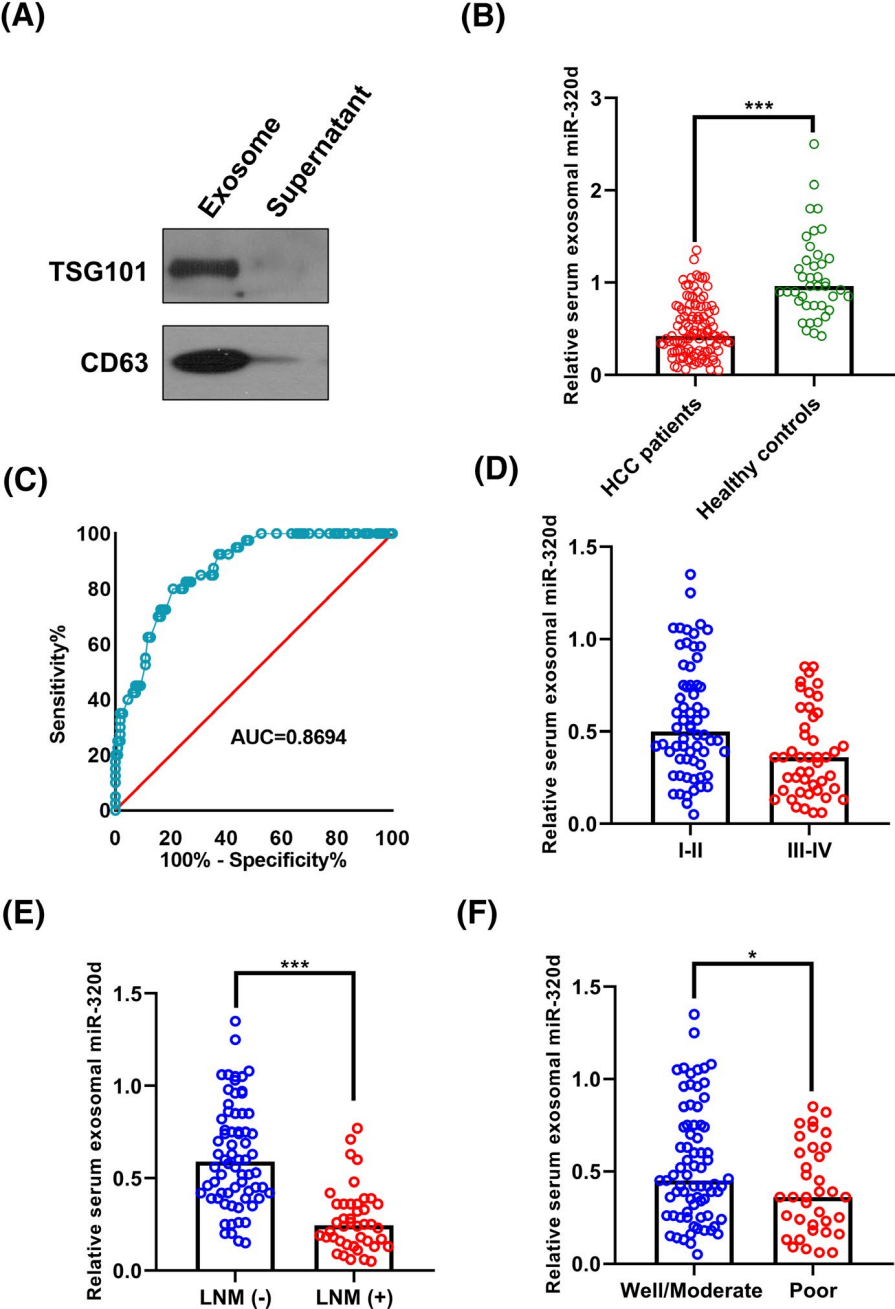
Serum exosomal miR‐320d was decreased in patients with HCC. A, TSG101 and CD63 were enriched in the exosomes isolated from serum sample. B, The expression level of serum exosomal miR‐320d was significantly lower in HCC patients than in healthy volunteers. C, Serum exosomal miR‐320d effectively discriminated HCC patients from healthy controls. D‐F, The levels of serum exosomal miR‐320d were lower in HCC patients at the advanced stage, or with positive lymph node metastasis, or with poorly differentiated tumor grade

**Figure 2:**
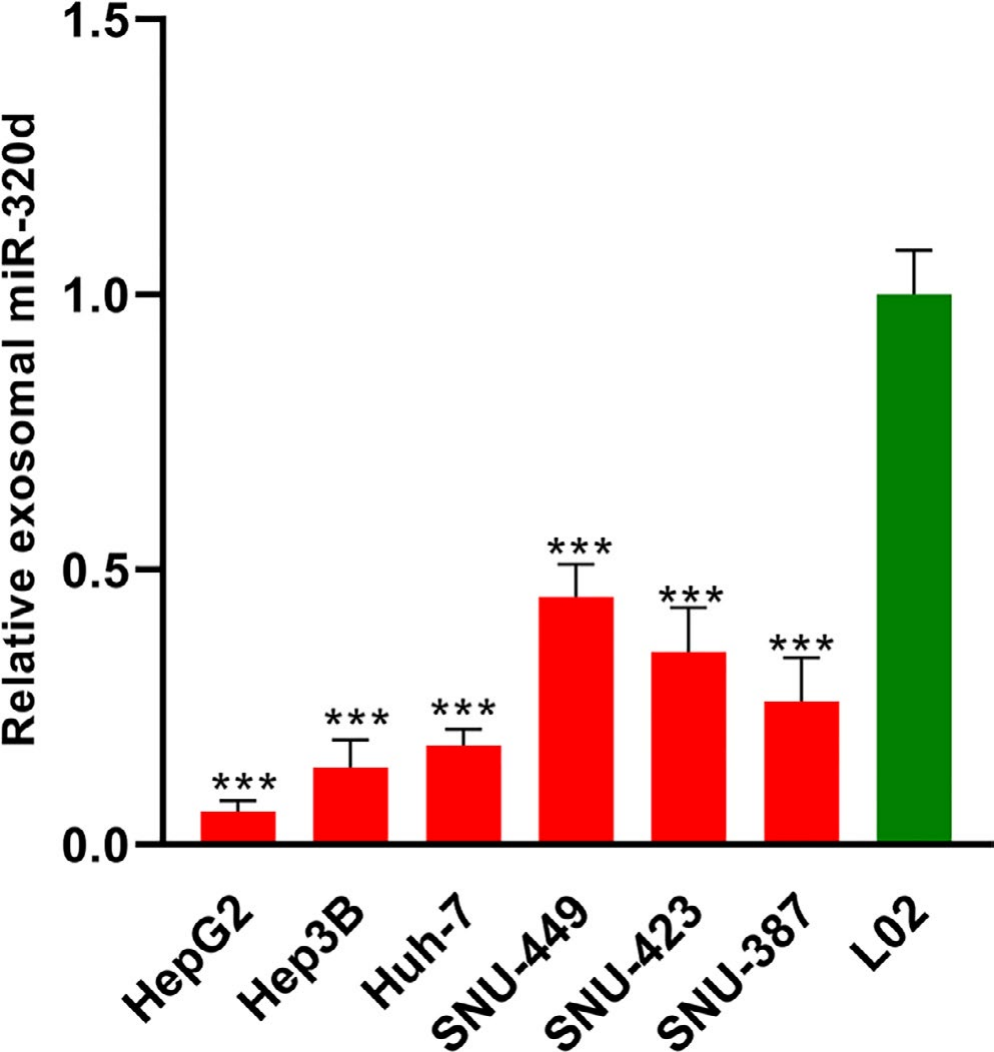
The expression level of exosomal miR‐320d was decreased in the culture medium from HCC cell lines compared to that from normal control cell line

The Western blot results showed that the exosomal markers (TSG101 and CD63) were highly enriched in the exosomes isolated from the serum samples (Figure 1A). Our qRT‐PCR results showed that the expression level of miR‐320d was significantly lower in patients with HCC than in healthy controls (***P < .001, Figure 1B). The ROC analysis revealed that serum exosomal miR‐320d was able to discriminate the HCC patients from the healthy controls with relatively satisfactory accuracy, and the AUC value was 0.8694 (*P* < .001, Figure 1C). Interestingly, we found that the serum exosomal miR‐320d levels were especially lower in HCC patients at the advanced stages (**P < .01, Figure 1D), or with positive lymph node metastasis (***P < .001, Figure 1E), or with poorly differentiated tumor grade (*P < .05, Figure 1F). In addition, the expression level of miR‐320d was also found to be downregulated in the culture media from HCC cell lines (HepG2, Hep3B, Huh‐7, SNU‐449, SNU‐423, and SNU‐387) compared to those from normal control cell line (L02; ***P < .001, Figure 2).

### 3.2 The association between serum exosomal miR‐320d and the clinicopathological parameter of HCC

The median level of serum exosomal miR‐320d was used to divide the HCC patients into high serum exosomal miR‐320d group (n = 53) and low serum exosomal miR‐320d group (n = 57). The Chi‐square analysis showed that serum exosomal miR‐320d was significantly associated with lymph node metastasis (*P* = .0045), TNM stage (*P* = .0056), and differentiation (*P* = .0297). However, it was not correlated with gender (*P* = .8938), age (*P* = .5256), tumor size (*P* = .2130), AFP level (*P* = .0901), and metastasis (*P* = .1968; Table 1).

### 3.3 Serum exosomal miR‐320d level was increased following surgical treatment

**Figure 3:**
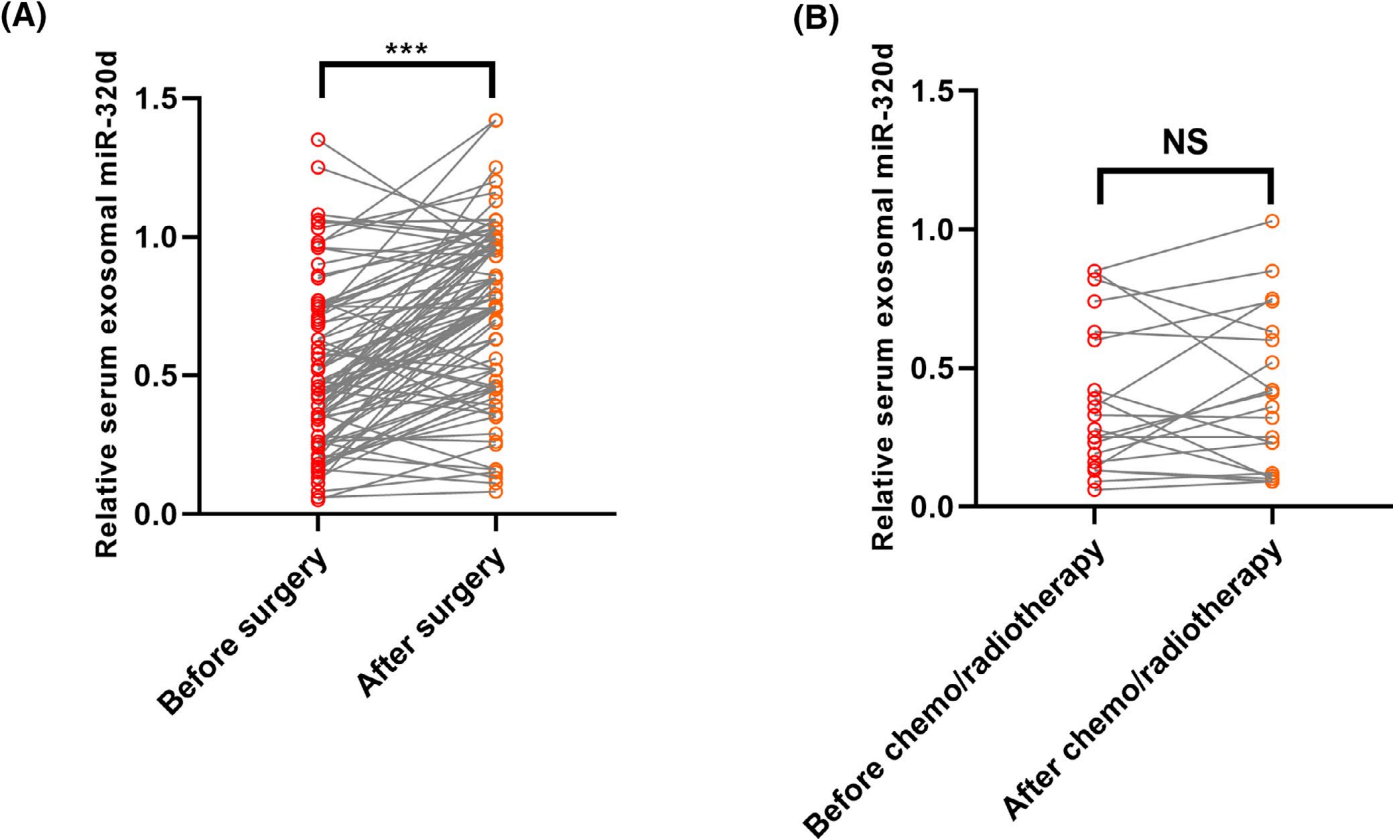
The therapeutic responses of serum exosomal miR‐320d. A, Serum exosomal miR‐320d was increased in HCC patients receiving surgical treatment. B, No significant difference was found for serum exosomal miR‐320d in HCC patients before and after the chemo/radiotherapy

A total of 89 HCC patients received the combination of chemoradiotherapy and surgery, and the remaining 21 patients received only the neoadjuvant chemoradiotherapy. We compared the exosomal miR‐320d level between the pre‐operative/pre‐chemoradiotherapy serum samples and post‐operative/post‐chemoradiotherapy serum samples (1 month). Our results showed that the expression level of serum exosomal miR‐320d was significantly increased in the HCC patients who received the surgical treatment (***P < .001, Figure 3A). However, no significant difference was found between pre‐chemoradiotherapy and post‐chemoradiotherapy serum samples (*P* > .05, Figure 3B).

### 3.4 Low serum exosomal miR‐320d was associated with unfavorable prognosis of HCC

**Figure 4:**
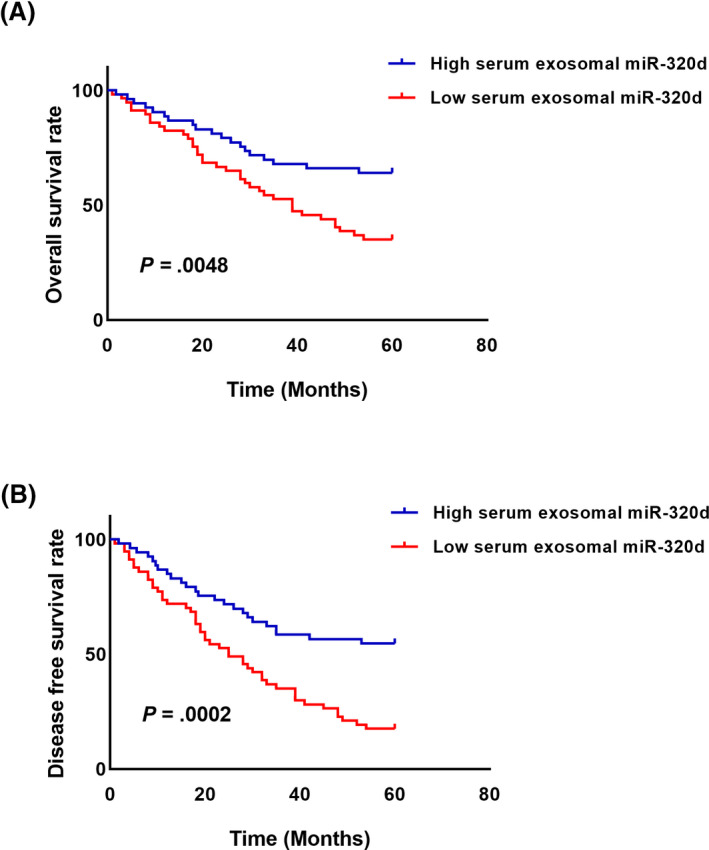
Lower serum exosomal miR‐320d was associated with unfavorable survival in HCC. A, The HCC patients in the low serum exosomal miR‐320d group had worse overall survival than those in the high serum exosomal miR‐320d group. B, Low serum exosomal miR‐320d was strongly associated with worse disease‐free survival rate in patients with HCC

Our survival analysis showed that the HCC patients in the low serum exosomal miR‐320d group had significantly shorter overall survival (*P* = .0048, Figure 4A) and disease‐free survival (*P* = .0002, Figure 4B) than the patients in the high serum exosomal miR‐320d group. The multivariate analysis revealed that serum exosomal miR‐320d was an independent prognostic factor (HR = 2.851, 95%CI = 1.18‐5.69, *P* = .021) for HCC (Table 2).

**Table 2:** Multivariate analysis of the independent prognostic factors of HCC

Variables	Multivariate analysis		
	HR	95% CI	*P*
Lymph node metastasis	4.75	1.52‐9.36	.002
TNM stage	3.06	1.30‐6.35	.016
Serum exosomal miR‐320d	2.85	1.18‐5.69	.021

### 3.5 Ectopic expression of exosomal miR‐320d inhibited the proliferation and invasion of HCC cells

**Figure 5:**
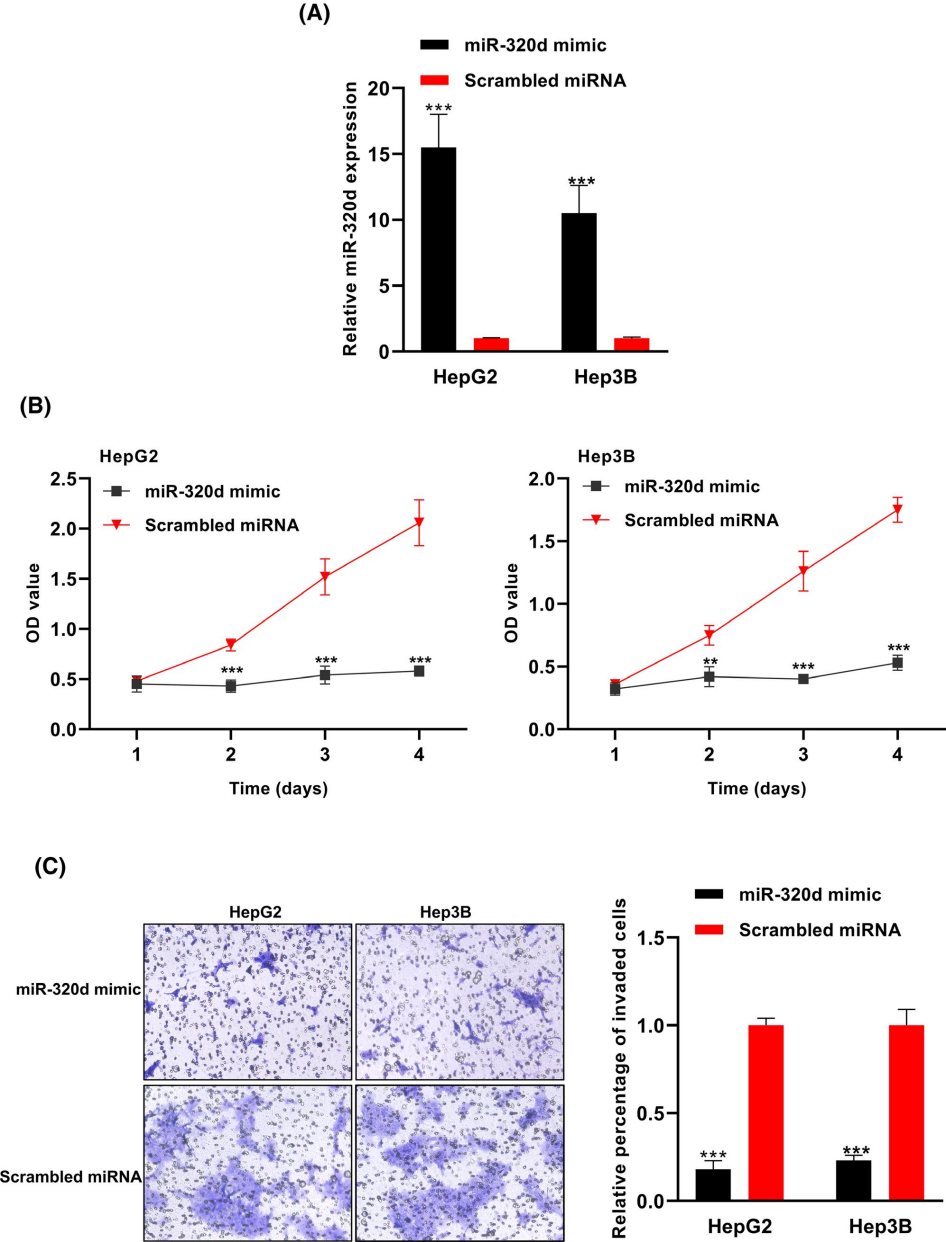
Overexpression of exosomal miR‐320d suppressed the proliferation and invasion capacity of HCC cells. A, The expression level of miR‐320d was significantly increased following miR‐320d mimic transfection. B, The OD values were lower in HCC cells with exosomal miR‐320d overexpression. C, The invasive capacity of HCC cells was lower in exosomal miR‐320d overexpression group compared with the control group

The miR‐320d mimic that transfected into the HCC cells significantly upregulated the expression level of miR‐320d (***P < .001, Figure 5A). The CKK8 assay showed that the OD values were significantly lower in miR‐320d mimic‐transfected cells compared to the scrambled miRNA‐transfected cells (**P < .01, ***P < .001, Figure 5B). The invasion assay showed that the miR‐320d overexpression cancer cells had lower invasive capacity than the control cells (***P < .001, Figure 5C).

**Figure 6:**
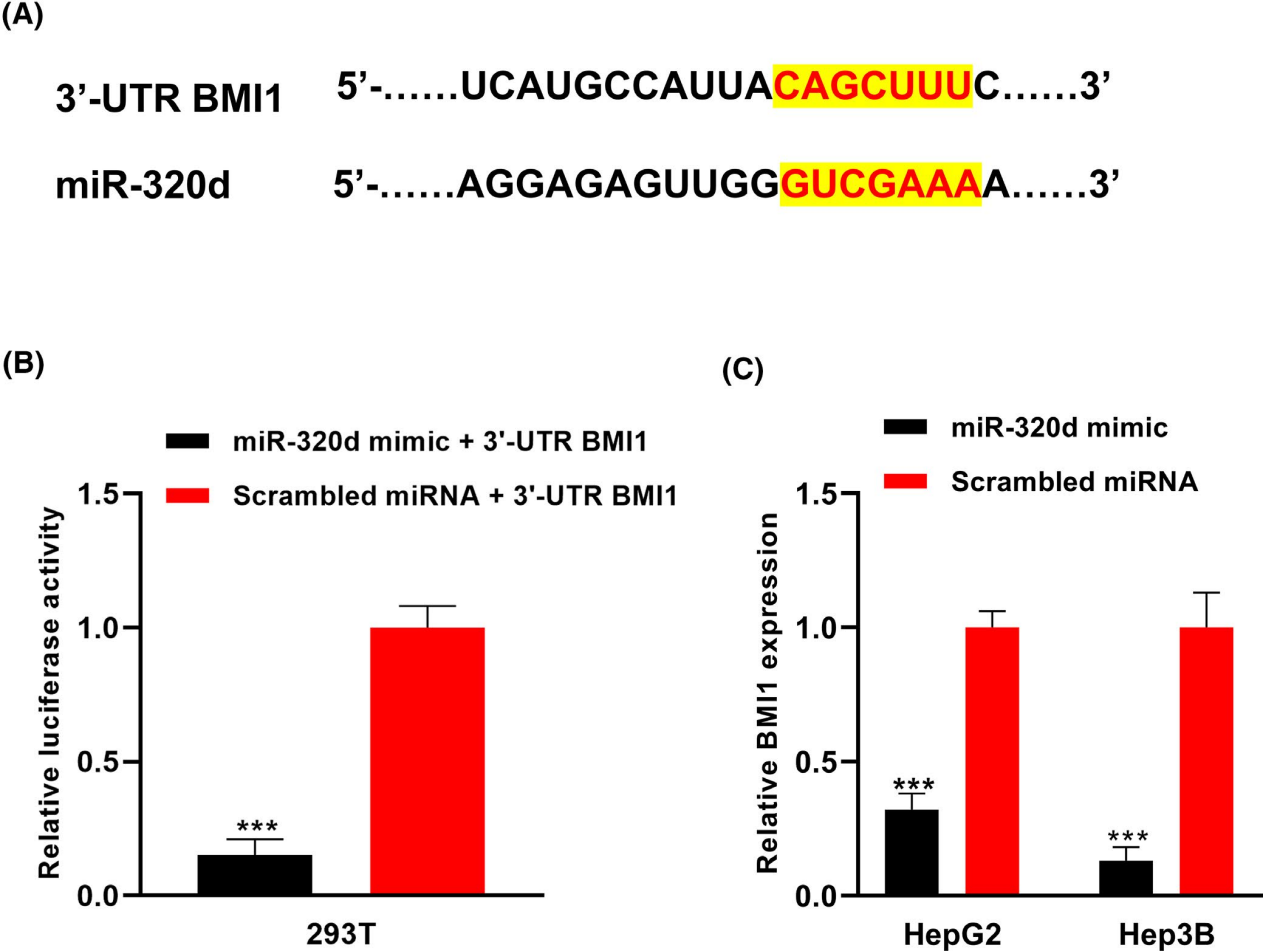
BMI1 was identified as the direct downstream target of miR‐320d in HCC cells. A, The 3′‐UTR region of BMI1 was highly complementary to the miR‐320d. B, The luciferase reporter assay showed that BMI1 was a direct target of miR‐320d. C, The expression level of BMI1 mRNA was significantly lower in HCC cells with exosomal miR‐320d overexpression

The bioinformatic analysis showed that the 3′‐UTR region of BMI1 was highly matched to the miR‐320d (Figure 6A). The luciferase reporter assay showed that the relative luciferase activity was lower in miR‐320 mimic group compared to the control group in 293t cells (***P < .001, Figure 6B). The qRT‐PCR validation experiment revealed that the expression levels of BMI1 were lower in the miR‐320 mimic group compared to the control group in the HCC cell lines (***P < .001, Figure 6C).

## 4 DISCUSSION

In this study, our results showed that the expression level of exosomal miR‐320d was significantly lower in the serum samples of HCC patients and the culture medium of HCC cell lines compared to their respective controls. Serum exosomal miR‐320d was effective to discriminate the HCC patients from healthy controls. In addition, its level was remarkably increased in those patients receiving surgery. Moreover, reduced serum exosomal miR‐320d was significantly associated with unfavorable clinicopathological parameters including advanced tumor stage, positive lymph node metastasis, and poor differentiation tumor grade. HCC patients with lower serum exosomal miR‐320d had dramatically shorter OS and DFS than those with higher serum exosomal miR‐320d. Low serum exosomal was identified to be an independent unfavorable prognostic factor for HCC. Finally, enforced expression of miR‐320d suppressed the proliferation and invasion of HCC cells, and BMI1 was demonstrated to be a direct target of miR‐320d. Collectively, these data suggested that serum exosomal miR‐320d was a good candidate biomarker for the diagnosis and prognosis prediction of HCC.

Exosomes secreted from the cancer cells contained the contents similar to that of the parental cells. They transferred the substances including miRNAs between cancer cells to create a microenvironment facilitating the initiation and progression of HCC.^16^ miRNAs are remarkably stable in the biofluids such as serum, plasma, and urine.^17^, ^18^ In addition, the biogenesis of exosome was increased in tumor and the cancer cells secreted many more exosomes compared to normal cells.^19^ Therefore, detecting tumor cell–derived exosomal miRNAs in the serum samples has potential clinical value for HCC diagnosis and prognosis.

Consistent with our results, the expression level of plasma miR‐320d was significantly reduced in patients with adenoma or colorectal cancer (CRC) patients compared to the healthy controls, suggesting circulating miR‐320d was promising for early diagnosis of CRC.^20^ Cancer stem cells (CSCs) are subpopulations of cancer cells driving tumor growth and heterogeneity.^21^ The miR‐320d level was remarkably lower in CD133‐positive colon CSCs than in the CD133‐negative controls,^22^ indicating miR‐320d might play a key role in maintaining stemness and tumorigenicity of CSCs. The expression levels of miR‐320 family including miR‐320a, miR‐320b, miR‐320c, miR‐320d, and miR‐320e were significantly reduced in colorectal adenoma and submucosal invasive carcinoma tissues as well as CRC cell lines. In addition, ectopic expression of miR‐320d inhibited the proliferation of CRC cells.^23^ Similarly, the expression of miR‐320d was downregulated in glioma tissues and cell lines. Overexpression of miR‐320d inhibited the cell growth, migration and invasion, and induced cell apoptosis as well as cell cycle arrest in glioma cells.^24^ Large B‐cell lymphoma patients with lower miR‐320d expression level suffered worse progression‐free survival and overall survival than those with higher miR‐320d expression level. What's more, ectopic expression of miR‐320d suppressed the proliferation of cancer cells, and *vice versa*.^25^ Interestingly, the expression level of circulating miR‐320d was found to be increased in patients with acute myeloid leukemia, indicating that miR‐320d might play an oncogenic role in AML.^26^ These findings suggest that the biological functions of miR‐320d are largely dependent on the cellular context and the downstream targets it affects.

One limitation of the current study was the relatively small sample size, and further studies with larger cohort are needed to be performed to confirm our findings. In addition, the molecular mechanism for the tumor‐suppressive role of exosomal miR‐320d in HCC warrants further exploration. Deregulation of circulating miR‐320d has also been reported in other diseases such as diabetes, acute stroke, and infant respiratory syncytial virus infection.^27^, ^28^, ^29^ Therefore, combination of serum exosomal miR‐320d and currently known biomarkers might contribute to the diagnosis and prognosis prediction of HCC.

In conclusion, we have demonstrated that the expression level of serum exosomal miR‐320d is reduced in HCC patients, and its downregulation is associated with unfavorable prognosis. Thus, it might serve as a promising biomarker for the diagnosis and prognosis prediction of HCC.

## CONFLICT OF INTEREST

None
